# FLYWCH1, a Multi-Functional Zinc Finger Protein Contributes to the DNA Repair Pathway

**DOI:** 10.3390/cells10040889

**Published:** 2021-04-13

**Authors:** Sheema Almozyan, James Coulton, Roya Babaei-Jadidi, Abdolrahman S. Nateri

**Affiliations:** 1 Cancer Genetics & Stem Cell Group, BioDiscovery Institute, Division of Cancer and Stem Cells, School of Medicine, University of Nottingham, Nottingham NG7 2UH, UK; ttxsa97@exmail.nottingham.ac.uk (S.A.); jamescoulton1996@gmail.com (J.C.); 2 Respiratory Medicine, School of Medicine, University of Nottingham, Nottingham NG7 2UH, UK; mszrb3@exmail.nottingham.ac.uk

**Keywords:** FLYWCH1, Cys2-His2 (C2H2)-type zinc-finger, DNA-damage response, γH2AX, ATM, p53, CRC

## Abstract

Over recent years, several Cys2-His2 (C2H2) domain-containing proteins have emerged as critical players in repairing DNA-double strand breaks. Human FLYWCH1 is a newly characterised nuclear transcription factor with (C2H2)-type zinc-finger DNA-binding domains. Yet, our knowledge about FLYWCH1 is still in its infancy. This study explores the expression, role and regulation of FLYWCH1 in the context of DNA damage and repair. We provide evidence suggesting a potential contribution of FLYWCH1 in facilitating the recruitment of DNA-damage response proteins (DDRPs). We found that FLYWCH1 colocalises with γH2AX in normal fibroblasts and colorectal cancer (CRC) cell lines. Importantly, our results showed that enforced expression of FLYWCH1 induces the expression of γH2AX, ATM and P53 proteins. Using an *ATM*-knockout (*ATM*^KO^) model, we indicated that FLYWCH1 mediates the phosphorylation of H2AX (Ser139) independently to ATM expression. On the other hand, the induction of DNA damage using UV-light induces the endogenous expression of FLYWCH1. Conversely, cisplatin treatment reduces the endogenous level of FLYWCH1 in CRC cell lines. Together, our findings uncover a novel FLYWCH1/H2AX phosphorylation axis in steady-state conditions and during the induction of the DNA-damage response (DDR). Although the role of FLYWCH1 within the DDR machinery remains largely uncharacterised and poorly understood, we here report for the first-time findings that implicate FLYWCH1 as a potential participant in the DNA damage response signaling pathways.

## 1. Introduction

Human cells are continuously under the challenge of different endogenous and exogenous stress, ultimately resulting in DNA damage. The maintenance of genome stability is of paramount importance for cell viability. Thus, cells have evolved multiple pathways, including the DNA damage response (DDR) pathway, to preserve genomic integrity and function [1,2]. The molecular mechanisms governing these processes are detailed and complex, with an immense interplay between the various signaling pathways. Nevertheless, the cellular response to DNA damage and the efficiency of the repair machinery is the primary determinant of patients’ outcome following treatments, as the majority of antitumour therapies exploit DNA damage to target the rapidly dividing cancer cells. Therefore, understanding and identifying novel players that govern DNA repair process is crucial for improving antitumour therapies’ effectiveness and developing novel targeted strategies.

*FLYWCH1* is a newly characterised gene. Hitherto, few studies investigated the interplay of physiological and molecular mechanisms by which FLYWCH1 function. For instance, FLYWCH1-mediated transcriptional regulation was found to be particularly crucial for the cardiovascular system [3]. Another study also suggested that mutation variants in *FLYWCH1* might be deleterious and associated with familial mitral valve prolapse (MVP) in humans, implying the alteration of the *FLYWCH1* gene expression in the cardiovascular system [4]. More recently, we showed that overexpression of FLYWCH1 reduces the motility and increases cell attachment in colorectal cancer (CRC) cells via modulating Wnt/β-catenin signaling. Likewise, FLYWCH1-overexpression acute myeloid leukaemia (AML) cells increases the number of cells in G0/G1 arrest, while decreases the number of cells at S and G2/M transitions by repressing nuclear β-catenin activity [5,6].

Basically, human FLYWCH1 protein is a conserved nuclear protein with multiple FLYWCH-type zinc-finger domains. FLYWCH1 belongs to Cys2-His2 (C2H2)-type Zinc Finger protein family and has a putative nuclear localization signal (NLS) motif (KRAK) that closely resembles the classical NLS motif consensus sequences (K-R/K- X-K/R) (uniprot.org/uniprot/Q4VC44). In the past years, the roles of zinc finger domain-containing proteins in the DNA damage response and double-strand breaks (DSBs) repair have gained great attention [7]. Several proteins have emerged as key players in many cellular processes such as cell migration and DSB repair [8,9,10]. However, the C2H2 zinc finger domain is best known for its role in sequence-specific DNA-binding proteins as the zinc finger transcription factor or in protein–protein interaction involved in post-translation modification (including FLYWCH1) [6,11,12,13]. A C2H2 zinc finger protein does not function as a zinc finger nuclease protein, which directly play a role in the DNA repair pathway [14]. Therefore, the actual role of human FLYWCH1 protein is still unclear.

According to the expressional data obtained from the Human Protein Atlas (HPA; proteinatlas.org/ENSG00000059122-FLYWCH1/cell#human), FLYWCH1 is mainly localised to the nuclear bodies and can be localised to the nucleoplasm and cytosol. In fact, the localisation of proteins to DNA damage sites is believed to be a hallmark of participation in DNA damage and repair processes [15]. At the DNA double-strand breaks (DNA-DSB) site, several proteins such as gH2AX, ATM, 53BP1, RAD51 and the MRE11/RAD50/NBS1 complex accumulate and/or modified to form microscopically visible subnuclear foci [15,16,17]. Interestingly, we here uncover a significant similarity between DNA-damage formed foci and FLYWCH1 nuclear foci. We showed a direct effect of FLYWCH1 expression on the H2AX phosphorylation independent to ATM protein. Additionally, for the first time, we envisaged the regulation of endogenous FLYWCH1 under the various DNA-damage stimuli. With a further clinical investigation in the future, such knowledge may not only lead to a better understanding of the complicated DDR signalling mechanisms, but may also offer a new target for anticancer therapies.

## 2. Materials and Methods

### 2.1. Cell Lines

Four different immortalized human colorectal cancer cell lines were used in this study HCT116, SW480, SW620 and DLD-1. All cell lines were originally purchased from the American Type Culture Collection (ATCC). In addition, human skin fibroblast cells (TIG119 cells) were a gift from Dr Peter Gordon laboratory (CRUK London Research Institute, London, UK).

### 2.2. Cell Culture and Treatment

Cells were cultured in RPMI1640 medium (Sigma Aldrich, Gillingham, UK, R0883) supplemented with 10% foetal bovine serum (FBS, Sigma Aldrich, Gillingham, UK, F7524) and 2 mM l-glutamine (Thermo Fisher Scientific, Altrincham, UK, 25030-024). Cells were then grown in a humidified, 37 °C, 5% CO_2_ incubator. Cell lines were routinely tested to exclude mycoplasma contamination. Treatments with UV-light, cisplatin (Tocris, Bristol, UK) or 6-bromoindirubin-30-oxime (BIO) (Sigma Aldrich, Gillingham, UK) were performed at indicated time points and concentrations and compared with control DMSO.

### 2.3. Generation of ATM Knock-Out Cell Lines

ATM-gRNA-CRISPR plasmid construct was generated by cloning ATM gRNA (TGGCACCGAGTCGGTGCTTTTT) [18] into pSpCas9 (BB)-2A-Puro (pX459) (Addgene plasmid ID 48139) backbone vector expressing puromycin resistance gene as a selection marker. The plasmid DNA was amplified into competent bacterial cells, and DNA was extracted using (medi-prep) kit (Qiagen, Manchester, UK). TIG119 cells were transfected with 5 µg of pLVX-puro lentivector constructs, using PEI (4 µL/1 µg) transfection reagent. Forty-eight hours later, transfected cells were selected by introducing 3 µg/mL of Puromycin (Sigma-Aldrich, Gillingham, UK, P8833) into the culture medium every 48 h for 7–10 days or until the complete death of un-transfected control cells. Surviving clones were screened for expression levels of ATM by immunoblotting and qPCR. Disruption of ATM locus was confirmed by Sanger sequencing.

### 2.4. Generation of Cell Lines Transiently Overexpressing FLYWCH1

An appropriate number of cells were seeded and allowed to reach 70% confluency to achieve optimum transfection efficiency. Cells were transiently transfected to overexpress GFP-tagged FLYWCH1 using Lipofectamine-2000 (Invitrogen Loughborough, UK). After 48 h, transfection efficiency was analysed by visual assessment of GFP expression using fluorescence microscope and cells were then processed for transcriptional analysis, mRNA or protein extraction.

### 2.5. Immunofluorescence

Samples were fixed in 4% PFA (Paraformaldehyde) for 30 min at RT, permeabilized using 0.1% BSA, 0.1% Triton X-100, 0.05% Tween-20 in PBS, blocked 3% BSA (Bovine Serum Albumin prepared in PBS) for 1 h at RT. Slides stained overnight with appropriate primary antibody concentration diluted in 2%BSA-PBS at 4 °C, followed by staining with secondary antibodies and imaging on a Zeiss LSM florescence microscope. Samples were counterstained with DAPI.

### 2.6. Western Blotting

Protein extracts were loaded into 4–20% or 10% Mini-PROTEAN TGX Precast Protein Gels (Bio-Rad, 4561096). Then, it was transferred to polyvinylidene difluoride PVDF membrane for 30 min at 15 V using a semi-dry electrophoretic transfer cell (Bio-Rad, Watford, UK). Blots were probed with the recommended concentration of primary Ab (1:500–1:1000) except for FLYWCH1 (1:200) was used (Prestige Antibodies, Sigma-Aldrich, Gillingham, UK, HPA041001). As suitable, antibodies against β-actin or β-tubulin were used as a loading control.

### 2.7. RNA Isolation and Quantitative PCR (qPCR)

Total RNA was extracted by TRIZOL reagent (Sigma-Aldrich) or by using the RNeasy Mini Kit (Qiagen) according to the manufacturer’s instructions. The cDNA synthesis was obtained by using PrimeScript Reverse Transcriptase (TAKARA, Saint-Germain-en-Laye, France) following the manufacturer’s instructions. A quantitative real-time polymerase chain reaction was performed based on SYBR green (Thermo Fisher Scientific, Altrincham, UK). All primers used are listed in Supplementary Table S1.

### 2.8. Statistical Analysis

Statistical analyses were obtained by Student’s *t*-test using GraphPad Prism 8 software or Microsoft Office Excel. For image analysis, Fiji (Image-J) software was used. The statistically tested experiments were repeated three independent times, and the results are shown as mean ± standard deviation (STDEV). The *p*-value ≤ 0.05 was considered statistically significant unless otherwise stated.

## 3. Results

### 3.1. FLYWCH1 Is Localised in Nuclear Speckles and Colocalised with γH2AX

We recently characterised and addressed the effect of *FLYWCH1* expression alterations, both knockdown and overexpression, on cell phenotype and biology. Our data suggested that alterations of FLYWCH1 expression influence the cell–cell attachment, migration, proliferation, and metastasis [5,6]. However, before this study, nothing was known about the role of FLYWCH1 in the DNA damage pathway. Thus, a preliminary idea on the genes that most likely to share function with FLYWCH1 based on their interactions was first created by employing the available proteomic prediction tools: GeneMANIA [19] (http://genemania.org, accessed on 13 April 2021) and STRING [20]. Our screening revealed high similarities and strong potential interactions of FLYWCH1 with proteins involved in DNA damage and repair, such as breast cancer type 1 susceptibility protein (BRCA1) and p53 binding protein (53BP1) (Supplementary Figure S1). Additionally, evidence from experimental protein–protein interaction reported in [21] has indicated potential interaction of FLYWCH1 with MDC1 protein involved in DNA damage and repair. Hereafter, several colocalisation studies of FLYWCH1 with crucial mechanisms involved in forming nuclear foci (such as DNA damage foci, chromatin foci and RNA foci) were carried out. For this, normal human skin fibroblast (TIG119) and CRC (HCT116) cell lines were used. Cells were transiently transfected with GFP-tagged FLYWCH1 and immunostained with the following antibodies for nuclear speckles markers: γH2AX, 53BP1, Drosha, HP1BP3 and HP1α. γH2AX encodes the phosphorylated form of H2AX (Ser139), a biomarker for DNA double-strand breaks (DSBs) [16]. 53BP1 encodes tumour suppressor p53 binding protein 1, an important DNA damage response factor [15]. Drosha is a double-stranded RNA-specific ribonuclease (RNase) III and a subunit of the microprocessor protein complex, contributes to DDR activation by generating small non-coding RNAs [22]. HP1BP3 (heterochromatin protein 1 binding protein 3) is a novel chromatin-binding protein [23]. HP1α, heterochromatin protein 1 isoform α, the main factor responsible for heterochromatin maintenance and gene silencing and is necessary for the binding of the main DNA damage-related protein 53BP1 at DNA repair foci [24].

**Figure 1:**
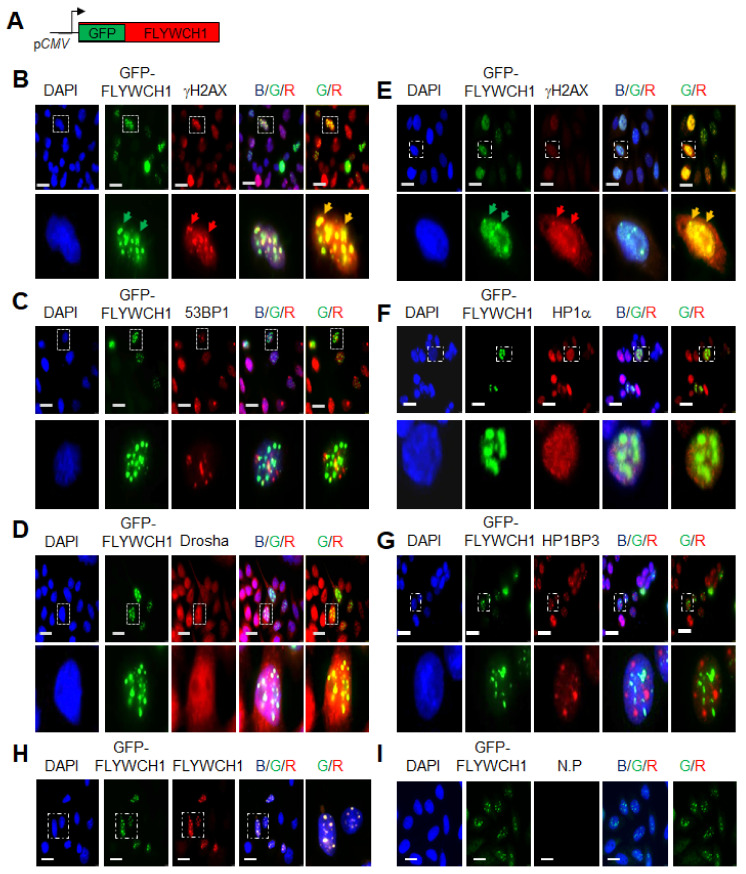
Immunostaining shows colocalisation of GFP-FLYWCH1 with γH2AX in TIG119 and HCT116 cells. (**A**) GFP-tagged FLYWCH1 transfected cells were fixed and stained with (**B**), (**E**) γH2AX, (**C**) 53BP1 and (**D**) Drosha. (**F**) HP1a, (**G**) HP1BP3, (**H**) anti-FLYWCH1 antibody staining confirm GFP-tagged FLYWCH1 exogenous expression (positive control) and (**I**) negative controls (N.P., no primary antibody). Arrowheads in (**B**,**E**) show representative colocalised FLYWCH1 and gH2AX foci. Dotted boxes indicate enlarged cell/s. Magnification, 100×. Scale bars, 7.5 μm.

The initial colocalisation study with γH2AX, 53BP1 and Drosha in TIG119 cells indicated a colocalisation of GFP-FLYWCH1 with γH2AX (Figure 1A), but not Drosha or 53BPI (Figure 1B,C), which were therefore excluded in HCT116 cells (Figure 1, right panel). Additionally, results showed that GFP-FLYWCH1 does not colocalise with HP1BP3, HP1a (Figure 1F,G), but γH2AX in FLYWCH1-expressing cells in HCT116 (Figure 1E). Unexpectedly, apart from the colocalisation with γH2AX, an upregulation of γH2AX was noticed in FLYWCH1-expressed cells (GFP^+^) in both normal and cancer cell lines (Figure 1A,E).

The colocalisation and steady induction of γH2AX in FLYWCH1-expressing cells has raised the question regarding the possible roles of FLYWCH1 in the DNA-damage response processes. While the minor histone H2A variant is a biomarker for DNA double-strand breaks (DSBs), the phosphorylation of H2AX, at ser-139, is one of the most well-established chromatin modifications linked to DNA damage and repair [25]. Although the contribution of FLYWCH1 in response to DNA damage is unknown, we aimed to understand the effects of DNA damaging agents (such as UV and chemotherapeutic agents) on the endogenous level and pattern of FLYWCH1 expression in normal fibroblasts versus CRC cells. At the same time, attempts were next aimed to investigate how FLYWCH1 mediates its effect on H2AX and study whether it requires DNA damage and DSB break (DNA-damage dependent).

### 3.2. The Effects of UV-Radiation on FLYWCH1 Expression in Normal vs. CRC Cells

**Figure 2:**
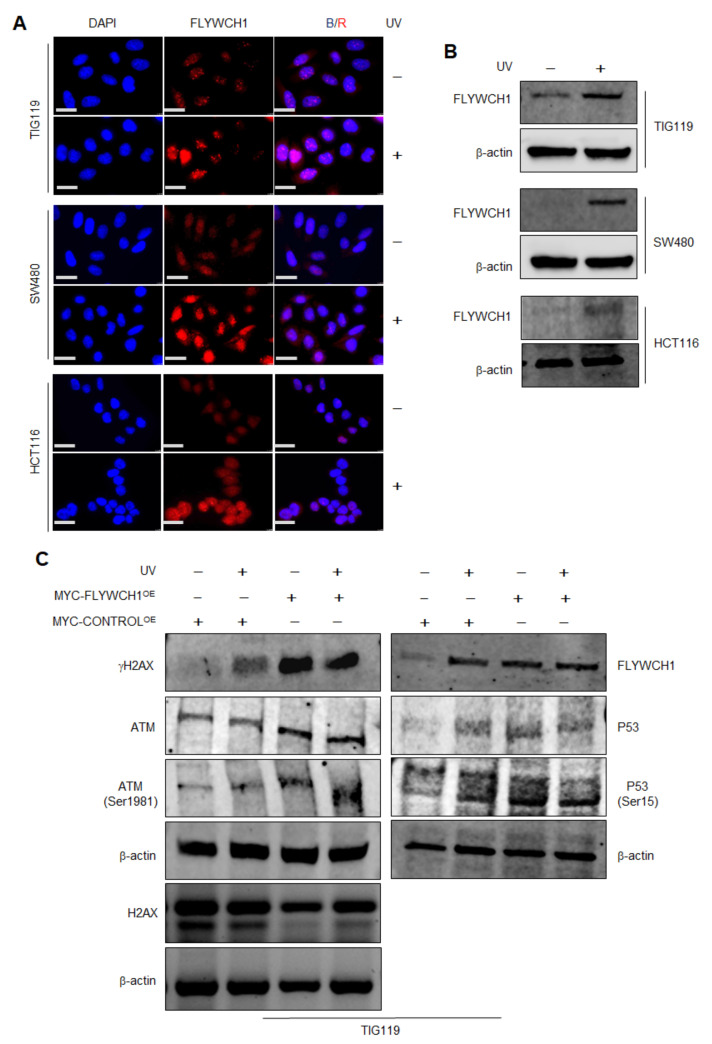
The regulation of FLYWCH1 expression in UV-mediated DNA damage. (**A**) Immunofluorescence (IF) staining showing the effect of UV on the expression level and pattern of FLYWCH1, Magnification, 100×, Scale bars: 7.5 μm. (**B**) Western Blotting (WB) analysis confirming the upregulation of FLYWCH1 protein in UV treated cells. Cells were seeded on coverslips for IF or dishes for WB, synchronized by starvation in serum-free medium before treatment with UV (50 mJ/cm^2^) (**C**) WB analysis demonstrates the recruitment of DDR proteins in response to UV exposure under normal versus enforced FLYWCH1 expression. Protein lysates (120 μg) extracted from UV-treated, and untreated fibroblasts were immunoblotted with γH2AX (ser139), ATM, ATM (ser1981), FLYWCH1, p53, p53(ser15) and H2AX antibodies.

UV light is a classical DNA-damage agent that induces H2AX phosphorylation [26]. Here, we examined the effect of UV-light on the endogenous expression of FLYWCH1 in different cell lines via Immunofluorescence (IF) staining and Western Blotting (WB) analysis. Remarkably, IF staining of endogenous FLYWCH1 in TIG119 and CRC cells revealed a clear induction, but no changes on the FLYWCH1 localisation under UV-light (Figure 2A). Furthermore, the UV-mediated induction of endogenous FLYWCH1 was confirmed by WB analysis (Figure 2B). The activation of the ATM/ATR signalling pathway in response to UV-induced DNA damage was validated by monitoring the foci formed by H2AX and ATM before and after UV irradiation by IF staining (Supplementary Figure S2). Together these results suggest an upregulation of FLYWCH1 protein in response to UV-mediated DNA damage. In parallel, the impact of exogenous overexpressed-FLYWCH1 (FLYWCH1^OE^) expression on DNA damage targets was studied and compared to UV-mediated outcomes. For this, TIG119 GFP (control) and MYC-tagged-FLYWCH1-*IRES*-GFP (MYC-FLYWCH1^OE^) overexpressing cells (Supplementary Figure S3A–D) were treated or mock-treated with UV-light. Cells were then analysed by IF staining and WB for the expression of several DDR proteins and endogenous FLYWCH1 protein. The activation of the ATM/ATR signalling pathway in response to UV-induced DNA damage was confirmed by the induction of H2AX and ATM protein level before and after UV irradiation (Figure 2C). In line with colocalisation data, FLYWCH1^OE^ induced γH2AX protein expression. Interestingly, the induction caused by FLYWCH1^OE^ was similar or higher than that caused by UV treatment (Figure 2C, lane 3 vs. lanes 2 and 4). Likewise, FLYWCH1^OE^ cells showed an elevated level of ATM, p53 and p53(Ser15) proteins regardless of UV-treatment condition, suggesting a positive correlation between FLYWCH1 expression with γH2AX induction (ATM/ATR activation) (Supplementary Figure S4A–G).

Collectively, data implied a direct impact of FLYWCH1 expression on H2AX phosphorylation and foci formation. Since ATM/ATR pathway is the primary signalling pathway implicated in UV-mediated DNA damage [26], it can be predicted that the recruitment and activation of ATM/ATR kinases could be the central mediator to execute FLYWCH1’s effect on γH2AX. Accordingly, we next investigated the impact of FLYWCH1 expression on H2AX using ATM-deficient cell lines.

### 3.3. FLYWCH1-Mediated Induction of Phosphorylated H2AX Level Is Independent of ATM Protein

**Figure 3:**
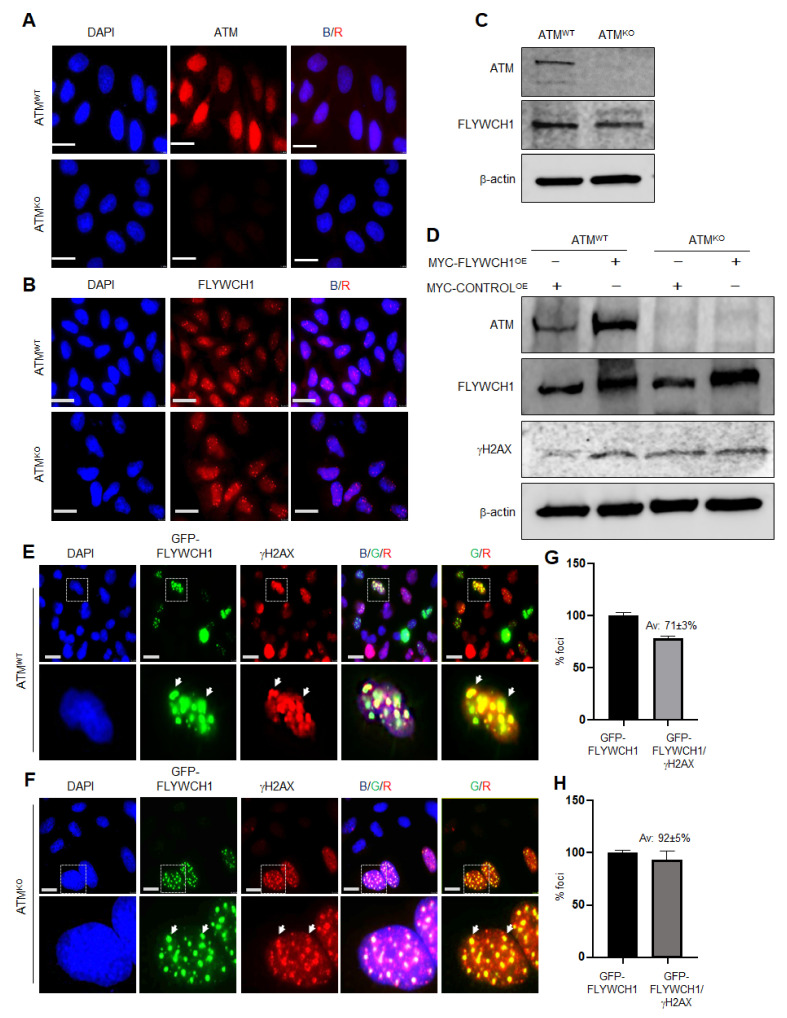
FLYWCH1-mediated induction of phosphorylated H2AX level is independent to ATM protein. (**A**,**B**) Immunofluorescence staining of the ATM protein and FLYWCH1 in TIG119, validating the depletion of ATM in the KO cells, and showing no underlying changes of FLYWCH1 level, magnification 100×. Scale bars: 7.5 μm (**C**) Western blot analysis of the un-transfected TIG119 cell lysates versus the ATM^KO^, validating the depletion of ATM in TIG119 cells and confirming the steady expression of FLYWCH1 in ATM^KO^ cells. β-actin was used as a control. (**D**) WB analysis and quantification of γH2AX under ATM depletion and FLYWCH1^OE^. (**E**,**F**) IF staining confirming the induction and colocalisation of FLYWCH1 (GREEN) with γH2AX foci (RED) in the absence of ATM protein, dotted boxes are magnified cells. Arrowheads in (**D**,**E**) show representative colocalised GFP-FLYWCH1 and gH2AX foci. Magnification: 100×, Scale bars: 7.5 μm. (**G**,**H**) percentage of FLYWCH1 foci that colocalise with γH2AX. Data are represented as mean fold change of two independent experiments, error bars indicate the standard deviation for each measurement.

Using CRISPR-Cas9 technology, we generated an ATM-knockout (ATM^KO^) model using TIG119 cells as explained in (M&M), and the cell line was validated for ATM depletion [18]. Afterwards, mRNA and protein expression of FLYWCH1 was studied in these KO cells by IF staining, WB and qPCR analysis. Initial screening showed no notable differences in the mRNA and protein level and/or the pattern of FLYWCH1 in ATM-depleted TIG119 cells (Figure 3A–C). Together the data signifying that ATM may not be crucial for FLYWCH1’s function. We thence questioned if FLYWCH1-mediated induction of γH2AX requires a functional ATM expression. For this, FLYWCH1 was overexpressed in both control (ATM^WT^) and ATM^KO^ cells and the γH2AX foci formation and its colocalisation with FLYWCH1 were examined. As demonstrated in Figure 3D and Supplementary Figure S4H, enforced-FLYWCH1 expression induced both ATM, γH2AX in the control cells.

Nevertheless, more importantly, overexpressing of FLYWCH1 in control and ATM^KO^ cells was sufficient to induce the foci formed by γH2AX irrespective to ATM expression (Figure 3E–H). Accordingly, our results implied that FLYWCH1 interplays with phosphorylation of H2AX in an independent of ATM activation. Therefore, ATM direct regulation of FLYWCH1 activity can be excluded in TIG119 cells. However, more information is required regarding the potential effects of ATM’s alterations and possibly other kinases, such as ATR and DNA-PKcS, on FLYWCH1 expression in CRC cells.

### 3.4. Cisplatin Treatment Reduced FLYWCH1 Protein Level in CRC Cells but Not in HCT116

**Figure 4:**
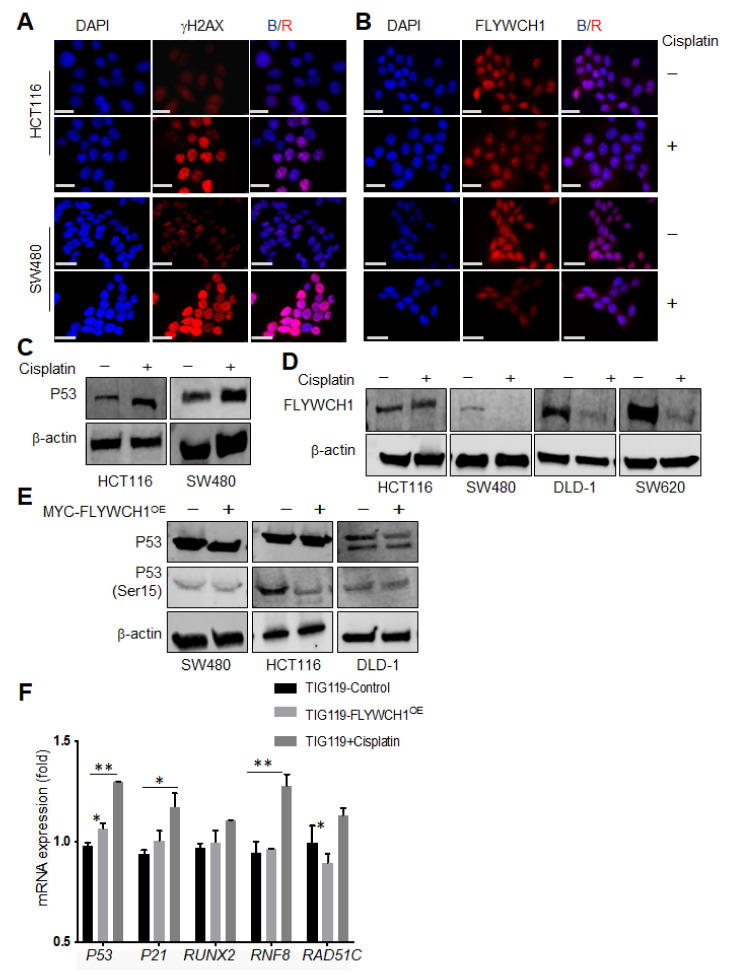
The regulation and role of FLYWCH1 in cisplatin-mediated DNA damage. (**A**,**B**) IF staining showing the induction and quick foci formation by γH2AX after 24 h of cisplatin treatment (Top panel), while the bottom panel showing the effect of cisplatin on FLYWCH1 protein level and pattern in CRC cells, Magnification: 100×, Scale bars: 7.5 μm. (**C**), WB analysis of the p53 level in HCT116 and SW480 cells following cisplatin treatment. (**D**) WB analysis showing the effect of cisplatin on FLYWCH1 expression in CRC cells. Cells were synchronised by overnight starvation in serum-free medium before treatment with 16 μg of cisplatin for 24 h, all CRC cells were maintained under the exact-similar conditions and passage number. Protein loading levels are monitored by probing for β-actin. (**E**) Expression and activity of p53 under ectopic expression of FLYWCH1 in CRC cell lines. HCT116, SW480 and DLD1 cells were transiently transfected with GFP-tagged FLYWCH1, protein lysates were immunoblotted with anti-p53, p53(Ser15) and anti-β-actin antibodies. (**F**) qPCR analysis of different DNA-damage targets under enforced FLYWCH1 expression and cisplatin treatment in TIG119 cells. mRNA expression was obtained by using the 2^-(∆∆Ct) method. Each sets of experiment are carried out in triplicate and repeated on two independent occasions. Data are mean ± SD (*, *p* < 0.05; **, *p* < 0.01).

Considering the effects of UV treatment on endogenous FLYWCH1 protein, the response to different DNA-damaging mediators was investigated by applying a chemotherapeutic (cisplatin) treatment strategy as another well-established type of DNA damaging agent. [27,28]. Most of the major DNA repair systems are involved in removing cisplatin-induced DNA damage [29]. Yet, despite the several players implicated in response to cisplatin treatment and drug sensitivity [28], a functional p53 is known to be dominant in the cellular response to cisplatin [30]. Therefore, employing cisplatin would provide information regarding the association of FLYWCH1 in p53-mediated cellular response. Cells were exposed to (16 μM) of cisplatin for 24 h and 48 h according to [31,32] to study the effect of cisplatin on the FLYWCH1. Cisplatin-mediated DNA damage was then validated by the activation of γH2AX (Figure 4A) [29], and the induction of p53 (Figure 4C). As shown in Figure 4A, cisplatin treatment caused a rapid formation of γH2AX foci within 24 h of treatment. It unexpectedly reduced the level of FLYWCH1 expression without significant changes in the pattern of expression in SW480 (Figure 4B, bottom panel). Unlike the UV+ effects, WB analysis showed that cisplatin drastically reduced FLYWCH1 protein level in TIG119 cells, and all p53-mutated CRC cell lines (Supplementary Table S2), except for HCT116 (p53^WT^) (Figure 4D). Furthermore, our qRT-PCR, of CRC cells treated with cisplatin showed no significant difference in mRNAs level (data not shown). In contrast, cisplatin treatment reduces protein level, possibly via proteasome degradation pathway (Supplementary Figure S5). These observations imply a p53-dependent notion of FLYWCH1 response to cisplatin-induced DNA damage.

Moreover, as indicated previously in (Figure 2B), ectopic FLYWCH1 expression induced both total and active form of p53 (Ser15) in TIG119 regardless of the UV-treatment condition. Therefore, the impact of enforced FLYWCH1 expression on p53 level and activity was also examined in CRC cells by WB analysis. Figure 4E revealed that ectopic expression of FLYWCH1 had no impact on the level/activity of p53 in SW480 or DLD-1 (p53^mut^) but reduced the active form of p53 (Ser15) in HCT116 (p53^WT^). Nevertheless, to ascertain a sparse yet informative perspective on the transcriptional signalling outputs activated under altered FLYWCH1 expression and cisplatin treatment, qPCR analysis of selected markers (p53, P21, RNF8, RAD51C and RUNX2) was carried out in TIG119 cells (Figure 4F).

In line with our hypothesis, genes affected by FLYWCH1 alteration were mainly p53-dependent targets and. As expected, cisplatin treatment showed an opposite effect to FLYWCH1^OE^, which aligned well with the observed downregulation of FLYWCH1 in cisplatin-treated cells (Figure 4B,D). Overall, as shown in Figure 4F, in cisplatin-treated cells, a substantial induction of p53, RUNX2, RNF8 and P21 was observed. These findings validate the activation of DNA damage and the requirements for our treatment condition. Remarkably, overexpressing FLYWCH1 in TIG119 cells increased the transcriptional activity of p53 and P21 (a direct target for P53 and associated with cell cycle arrest response [33]). While RNF8 (ATM-dependent target [34,35]) was not affected by FLYWCH1-expression, supporting the notion of FLYWCH1’s ATM-independent function. In addition, RUNX2 was slightly reduced by FLYWCH1^OE^ (RUNX2 plays a role in p53-mediated DNA damage response, repressing p53-dependent apoptotic cell death following DNA damage [36,37]). Likewise, RAD51C was significantly reduced by FLYWCH1-expression (which is crucial for the early and late homologous recombination (HR) stages and required for ATM-mediated phosphorylation of CHK2 [38]).

While initial results suggested a potential contribution of p53 in the mechanism by which FLYWCH1 acts and/or regulated in CRC in response to cisplatin-mediated DNA damage, still further analysis addressing the impact of p53 inhibition or depletion on FLYWCH1 will be needed to elucidate the association between the two proteins. Despite the substantial role of p53 in cisplatin-induced DNA damage response, increasing evidences suggest that several signalling pathways such as Akt, PKC and MAPKs (e.g., ERK, JNK and p38 MAPK) can regulate cisplatin sensitivity and cisplatin-induced apoptosis in p53-negative cells [29]. Thus, future experiments looking at different signalling pathways and their association with FLYWCH1 functions would provide an extensive evaluation of the role and regulation of FLYWCH1 under different circumstances.

## 4. Discussion

This study sheds light on the new potential role of FLYWCH1 protein in the DNA-damage response signalling pathways. Nevertheless, undoubtedly, the functions and regulation of FLYWCH1, particularly in cancer, could be modulated through multiple signalling pathways and are not restricted to Wnt signalling [6]. In this study, we were striving to uncover new faces of FLYWCH1, adding new insight into the function and biological significance of this protein. We showed FLYWCH1, under normal conditions, is a nuclear protein and endogenously expressed as nuclear foci. FLYWCH1’s nuclear speckles are highly similar to those proteins recruited under DNA damage (e.g., γH2AX).

When a cell encounters stress like elevated reactive oxygen species (ROS) levels, nutrient starvation or DNA damage, several nuclear proteins and markers are activated in order to partake in re-establishing cellular homeostasis [39]. It was, therefore, important to question the possible role of FLYWCH1 foci in the mechanisms involved in DNA-damage response and examine whether it can be recruited under DNA damage by considering the impact of DNA damaging agents on FLYWCH1 function, expression, and perhaps localisation within the cells.

To this end, several colocalisation studies were carried out, and the results revealed a consistent colocalisation of FLYWCH1 with γH2AX (Figure 1). The phosphorylation of H2AX (Ser139) plays a crucial role in DDR and is required for the assembly of DNA repair proteins at the sites of damage and activation of checkpoints protein [16]. Interestingly, we indicated that FLYWCH1 overexpression induced the foci formed by γH2AX, regardless of DNA damage. Results also showed that FLYWCH1 induces the expression of γH2AX in a similar way to UV-treatment, with slight effects on ATM and p53 (Ser15) proteins. The combination of UV and FLYWCH1 expression demonstrated a higher level of H2AX compared to UV-treatment only (Figure 2). Contrastingly, UV treatment induced the endogenous FLYWCH1 protein, and this was correlated with induction of DDR associated proteins (e.g., ATM and γH2AX), suggesting that FLYWCH1 could also be recruited under DNA damage.

UV-light recruits a DDR mechanism different from cisplatin, and therefore FLYWCH1 can be regulated via various upstream mediators depending on the type of DNA-damage lesions. ATM/ATR activation is the primary DNA damaging pathway responsible for responding to UV-induced DNA lesions. However, several pathways are involved in sensing cisplatin-induced DNA damage, including Akt, PKC and MAPKs (e.g., ERK, JNK and p38), and p53 [26,28,29,30,31,40,41]. Additionally, it is well-established that phosphorylation of H2AX in response to DSBs is mediated by PIKK family proteins, involved in both ATM/ATR and DNA-PKcs [25]. Thus, we hypothesised a potential contribution of FLYWCH1 in the recruitment, activation, ATM/ATR and subsequent phosphorylation of H2AX. Accordingly, the necessity of ATM in FLYWCH1’s functions was first examined using the ATM^KO^ model. Unexpectedly, experiments showed no direct effect of ATM-depletion on the endogenous FLYWCH1 expression or its action on H2AX (Figure 3). FLYWCH1 was still able to induce the γH2AX level, independently of ATM expression. Hence, it was suggested that FLYWCH1 mediates the phosphorylation of γH2AX in ATM independent manner and perhaps without DNA damage.

There is also increasing evidence for the induction of H2AX phosphorylation in conditions that do not involve DNA damage, such as serum starvation [42]. Hence, γH2AX induction does not always indicate the presence of DSBs, as H2AX can also be phosphorylated in a cell cycle-dependent manner [43]. This study [43] indicated that the DNA-PKcs/CHK2 pathway mediates the mitotic phosphorylation of H2AX in the absence of DNA damage. Accordingly, the foci formed by γH2AX under altered FLYWCH1-expression could be related to cell-cycle or other unknown reasons. However, little is known about the regulation of γH2AX in association with cell cycle progression or DNA-damage independent factors. Thus, the contribution of FLYWCH1 expression in γH2AX foci formation out of the DNA-damage context is still under question. Further experiments on the effects of FLYWCH1 on γH2AX at different stages of cell-cycle might provide more robust evidence on how FLYWCH1 functions, yet this is beyond the scope of the current study.

In keeping with the role of the ATM/ATR signalling pathway in UV-mediated DNA damage, our findings indicated the significance of ATM protein expression in the mechanism by which UV/cisplatin mediates their action on FLYWCH1. Nevertheless, the involvement of ATR protein cannot be excluded. Another intriguing observation in this study was the contrasting effects of different DNA damaging agents on the endogenous FLYWCH1 in CRC cells. Unlike UV treatment, cells treated with cisplatin showed a significant reduction in FLYWCH1 expression, where cisplatin treatment suppressed FLYWCH1 protein level in all CRC cells (P53^mut^) except for HCT116 (p53^WT^) (Figure 4). Several pathways are involved for sensing cisplatin-induced DNA damage, including Akt, PKC and MAPKs (e.g., ERK, JNK and p38 MAPK). However, the tumour suppressor protein p53 plays the most critical role in cisplatin-induced DNA damage response and known to be the primary mediator [27,44]. When cells receive DNA damage, p53 is quickly activated and induces cell cycle arrest and/or apoptotic cell death by transactivating its target genes implicated in promoting of cell cycle arrest and/or apoptotic cell death such as p21WAF1, BAX and PUMA [44]. A study has shown that the induction of apoptosis followed by genotoxic damage caused by cisplatin needs a functional p53 [30]. An additional but important player that influences response to cisplatin treatment is the activation of c-ABL, which requires the ATM protein, DNA-PK and a functional DNA mismatch repair response [28]. With all the above considerations, the potential role of p53 in regulating FLYWCH1’s response to cisplatin cannot be neglected.

Nonetheless, our data suggested no effect of FLYWCH1^OE^ on the level of total p53 in all three CRC cell lines (p53wt/mut). However, the phosphorylated form of p53 (Ser15) was reduced only in the HCT116 cells harbouring WT-p53 (Figure 4E). However, the total and phosphorylated form of p53 (Ser15) was increased in TIG119 cells with WT-p53 in response to UV treatment (Figure 2C, and Supplementary Figure S4). These data indicate that FLYWCH1 might need an intact p53 to facilitate its influence. Furthermore, a transcriptional analysis of several genes targeted by either p53/ATM and/or cell-cycle signalling mechanisms was considered under altered FLYWCH1 expression. Here, an apparent effect of FLYWCH1-expression was perceived on p53 and p53-putative targets (p21, RUNX2), but not ATM-targets (RNF8) (Figure 4F). Therefore, our results suggest a direct link between FLYWCH1 and p53-dependent DNA damage response, although addressing the impact of p53 gain/loss of function on FLYWCH1 expression would provide greater insight into their association.

In conclusion, we provided evidence supporting a novel role of FLYWCH1 in the DNA-damage response signalling pathways. Moreover, our results suggest a mechanism by which different DNA-damage stimuli act to regulate FLYWCH1 endogenously in normal and CRC cell lines. Future research studying the upstream mediators in regulating the FLYWCH1 response to DNA damage; and examining FLYWCH1 recruitment at the damage sites will provide a better understanding of FLYWCH1’s role in DNA damage/repair processes. While FLYWCH1’s role in DNA damage and repair is still unknown, our findings here suggest that enforced FLYWCH1 expression would trigger the induction and recruitment of DDRP, which does not involve ATM protein. In contrast, the response of endogenous FLYWCH1 to DNA damage requires a functional ATM protein. Such knowledge will expand our understanding of the various functions mediated by FLYWCH1, adding a novel C2H2 ZF-containing player in the DNA damage and repair processes.

Moreover, studying the contribution of FLYWCH1 in the formation of non-DNA damage foci by H2AX would also provide more information regarding the multi-functions of FLYWCH1. To sum up, we can speculate that FLYWCH1 interacts with core components of the DNA repair machinery. Thus, future work could help to disclose the exact contribution and other relevant roles of FLYWCH1 in this context.

## Data Availability

Not applicable.

## Supplementary Materials

The following are available online at https://www.mdpi.com/article/10.3390/cells10040889/s1, Figure S1: Predicted protein interaction network, Figure S2: γH2AX and ATM used as a positive control for UV light. Figure S3: Validation of MYC-tagged FLYWCH1 overexpressing using IF and WB assays. Figure S4: Quantitation of the changes in band intensities of WBs. Figure S5: The effect of cisplatin on FLYWCH1 protein expression in HCT116 and SW480 cell lines. Figure S6: Unprocessed WBs used in Figure 2. Figure S7: Unprocessed WBs used in Figure 3. Figure S8: Unprocessed WBs used in Figure 4. Table S1: List of primers and their sequences used in this study. Table S2: Colon cancer cell lines origins and gene mutations.

## Abbreviations

BRCA1	Breast cancer type 1 susceptibility protein
BSA	Bovine Serum Albumin
β-TRCP	Beta-Transducin Repeat-Containing Protein
C2H2	Cystein2-Histidine2
CRC	Colorectal Cancer
CRISPR	Clustered regularly interspaced short palindromic repeats
DAPI	4′,6-diamidino-2-phenylindole
DDR	DNA Damage Repair
DNA	Deoxyribonucleic acid
dH2O	Distilled Water
dsRNA	Double stranded RNA
DSB	Double-strand breaks
Drosha	Double-stranded RNA-specific ribonuclease (RNase) III
FLYWCH1	FLYWCH-type zinc finger 1
GFP	Green Fluorescent Protein
gRNA	Guide RNA
H2AX	Histone H2A variant
HDAC	Histone Deacetylases
HP1a	Heterochromatin protein 1 isoform a
HP1BP3	Heterochromatin protein 1 binding protein 3
HR	Homologous recombination
MMR	Mismatch repair
MSI	Microsatellite Instability
MSS	Microsatellite Stability
NER	Nucleotide excision repair
NHEJ	Non-homologous end-joining
NLS	Nuclear localization signals
ROS	Reactive Oxygen Species
UV	Ultraviolet
