# Supplementary material for: FLYWCH1, a Multi-Functional Zinc Finger Protein Contributes to the DNA Repair Pathway
Source: Cells. 2021 Apr 13;10(4):889. doi: 10.3390/cells10040889 (PMC8069811; doi:10.3390/cells10040889)
Supplement: Supplementary file 1 [file cells-10-00889-s001.pdf]

Supplemental Materials

FLYWCH1, a Multi-Functional Zinc Finger Protein Contributes to the DNA Repair Pathway

Sheema Almozyan, James Coulton, Roya Babaei-Jadidi, and Abdolrahman S. Nateri

Supplementary Materials: The following are available online at www.mdpi.com/xxx/s1, Figure S1: Predicted protein inter-action network, Figure S2: γH2AX and ATM used as a positive control for UV light. Figure S3: Validation of MYC-tagged FLYWCH1 over-expressing using IF & WB assays. Figure 4: Quantification of the changes in band intensities of WBs. Figure S5: The effect of cisplatin on FLYWCH1 protein expression in HCT116 and SW480 cell lines. Figure S6. Unprocessed WBs used in Figure 2. Figure S7. Unprocessed WBs used in Figure 3. Figure S8. Unprocessed WBs used in Figure 4. Table S1: List of primers and their sequences used in this study. Table S2: Colon cancer cell lines origins and gene mutations.

Supplementary Table 1.
List of primers and their sequences used in this study.

	**Primer sequences 5'- 3'**
**FLYWCH1**	Forward: CTGGATGCAGCCCCTCAGT<br>Reverse: TTGGCGGCACTTCCAGTAC
**HPRT**	Forward: AGATGTGATGAAGGAGATG<br>Reverse: GTGTCAATTATATCTTCCA
**B-actin**	Forward: GCGCGGCTACAGCTTCA<br>Reverse: CTTAATGTCACGCACGATTTCC
**RAD51C**	Forward: AAATGCAGCGGGATTTGGTG<br>Reverse: CCCAACTTCTTTGCTAAGCTCG
**RNF8**	Forward: TCATTGAGGCTGTCACCTTGA<br>Reverse: TGTCCTTCCGACAAATGGGG
**RUNX2**	Forward: GTCCCCGTCCATCCACTCTA<br>Reverse: GGTGGCAGTGTCATCATCTGAAA
**P21**	Forward: AGCTGAGGTGTGAGCAGC<br>Reverse: TTCTGACATGGCGCCTCC
**p53 (TP53)**	Forward: AAGTCTAGAGCCACCGTCCA<br>Reverse: GCAGTCTGGCCAATCCAGG

Supplementary Table 2.
Colon cancer cell lines origins and gene mutations.

Name	Cell line origins	TP53	KRAS	BRAF	PIK3CA	PTEN	MSI	CIMP
**HCT116**	Tissue: Colon<br>Disease: Colorectal carcinoma, primary tumour, Dukes' D	WT	p.G13D	WT	p.H1047R	WT	MSI	CIMP+
**DLD-1**	Tissue: Colon<br>Disease: Dukes' C, Colorectal adenocarcinoma.	p.S241F	p.G13D	WT	p.E545K<br>p.D549N	WT	MSI	CIMP+
**SW480**	Tissue: Colon<br>Disease:Dukes' B, Colorectal adenocarcinoma.	p.R273H;<br>p.P309S	p.G12V	WT	WT	WT	MSS	CIMP-
**SW620**	Tissue: colon; derived from metastatic site: lymph node<br>Disese: Dukes' type C, colorectal adenocarcinoma	p.R273H;<br>p.P309S	p.G12V	WT	WT	WT	MSS	CIMP-

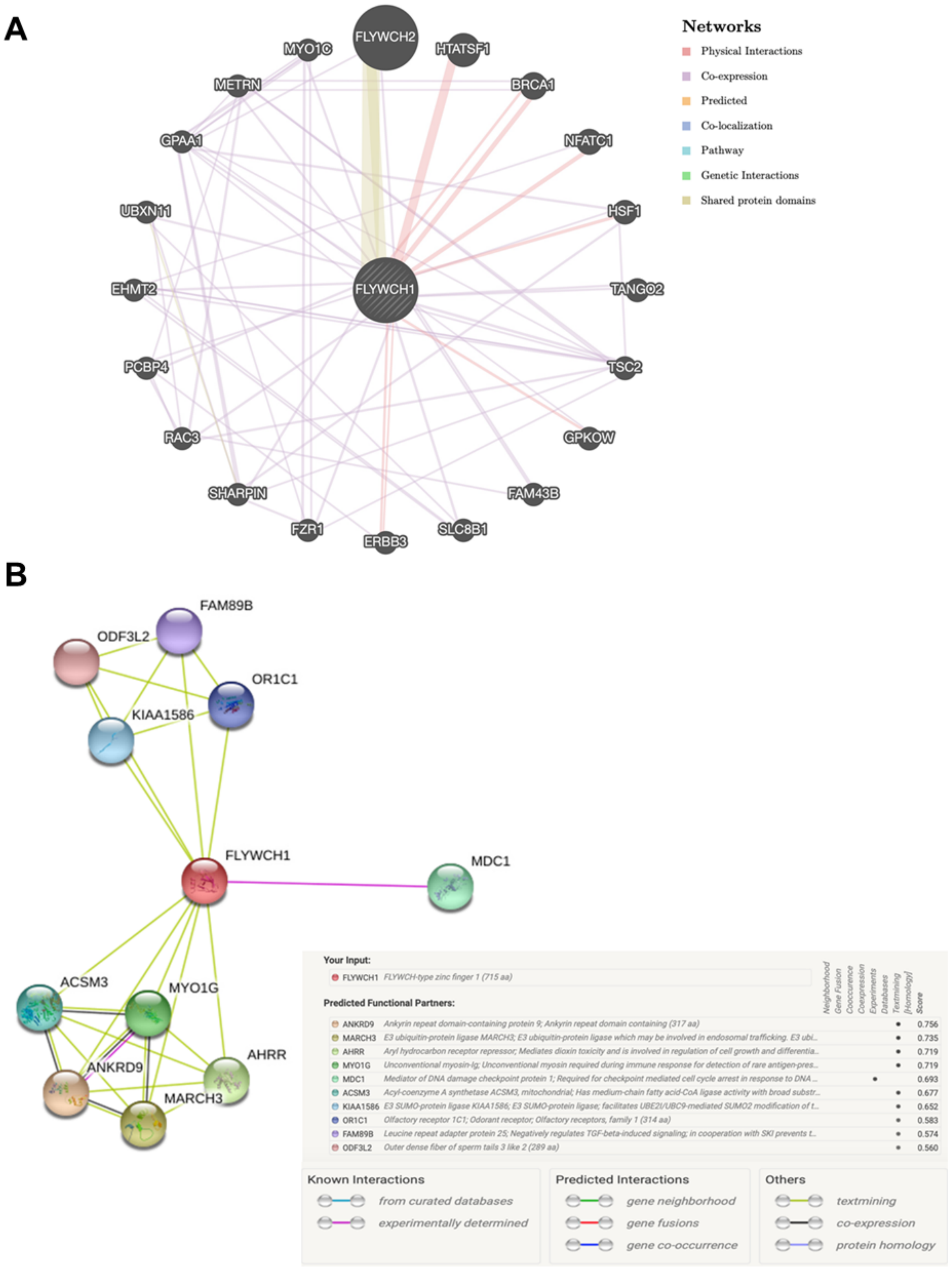

Supplementary Figure 1, Almozyan S et al.

Supplementary Figure 1. Predicted protein interaction network generated by A) GeneMANIA [1] software, the diagram indicates high similarities and strong potential interactions of FLYWCH1 with proteins involved in DNA damage and repair such as breast cancer type 1 susceptibility protein (BRAC1) and p53 binding protein (53BP1) based on data of gene co-annotation in the Gene Ontology biological function hierarchy. **B)** STRING software, the map indicates an experimental based potential

interaction of FLYWCH1 with MDC1 protein involved in DNA damage and repair based on experiments. Evidence from experimental protein-protein interaction reported in [2].

1. Franz, M., et al., *GeneMANIA update 2018.* Nucleic acids research, 2018. **46**(W1): p. W60-W64.
2. Gupta, R., et al., *DNA Repair Network Analysis Reveals Shieldin as a Key Regulator of NHEJ and PARP Inhibitor Sensitivity.* Cell, 2018. **173**(4): p. 972-988.e23.

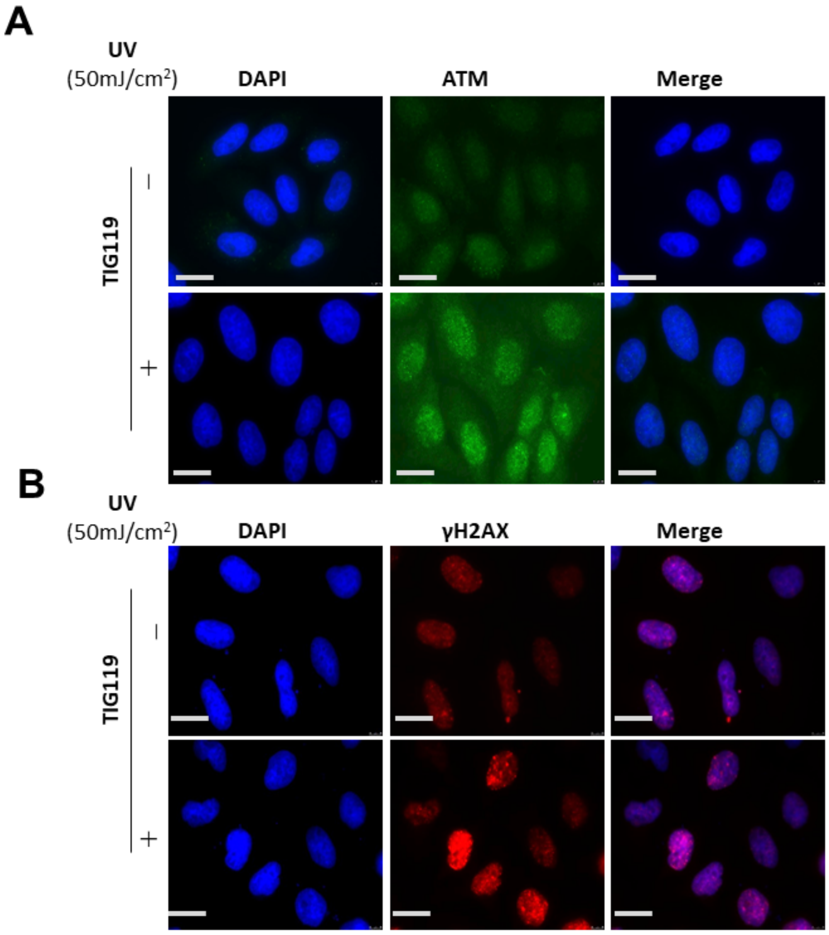

Supplementary Figure 2, Almozyan S et al.

Supplementary Figure 2. γH2AX and ATM used as a positive control for UV light. TIG119 cells were exposed to 50mJ/cm² UV light and immunostained with **A)** ATM and **B)** γH2AX. Images demonstrate the induction and foci expression pattern of γH2AX following UV treatment. Nuclei were detected with DAPI-blue fluorescent stain. Magnification, 100x. Scale bars: 7.5μm.

Supplementary Figure 3, Almozyan S et al.

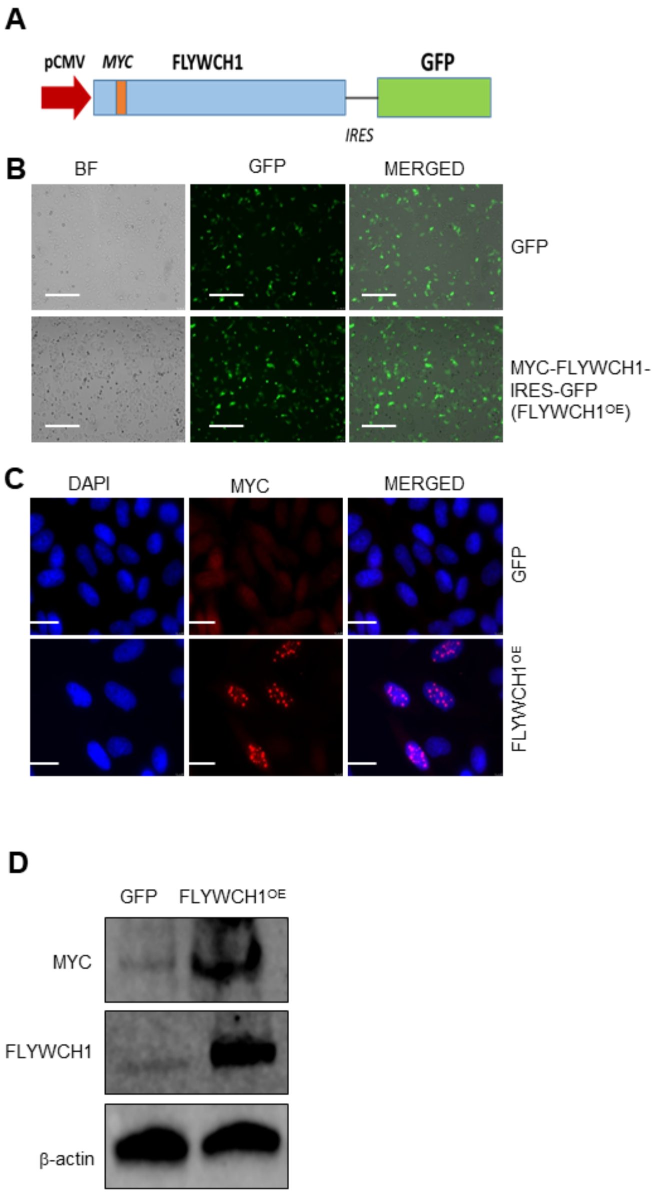

Supplementary Figure 3. Validation of MYC-tagged FLYWCH1 over-expressing using IF & WB assays. A) Schematic of the MYC epitope tagged FLYWCH1 – IRES- GFP construct. **B)** GFP expression of transfected cells indicated the efficiency of transfected cells. **C)** Validation of MYC-tagged FLYWCH1 expression using anti-MYC antibody. **D)** Validation of MYC-tagged FLYWCH1 expression using anti-FLYWCH1 antibody.

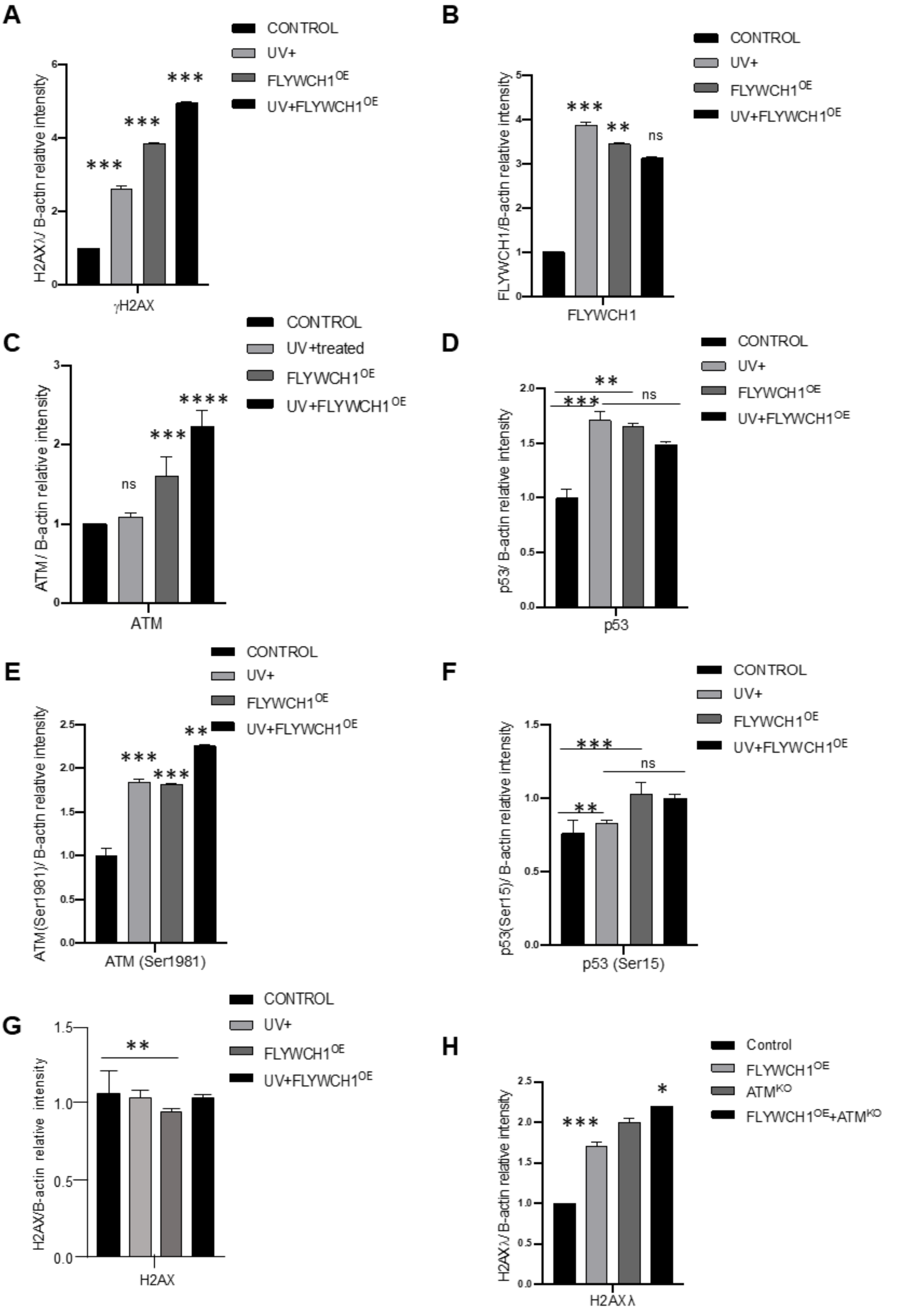

Supplementary Figure 4, Almozyan S et al.

Supplementary Figure 4. Quantitation of the changes in band intensities of WBs presented in Figure 2C and 4D. Histogram shows A) γH2AX, B) FLYWCH1, C) ATM, D) p53, E) ATM(Ser1981), F) p53(Ser53), G) H2AX presented in Figure 2C and H) γH2AX presented in Figure 4D, ratio band intensity following normalization with β-actin. Data are represented as mean fold change of three independent experiments, error bars indicate the standard deviation for each marker measurement.

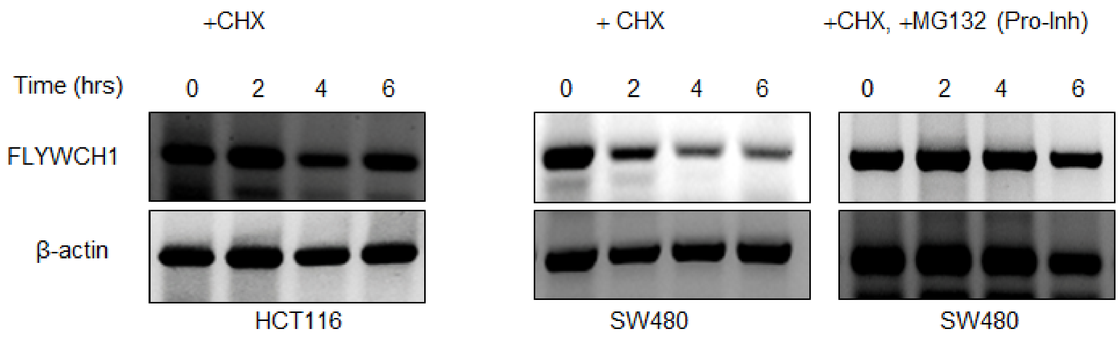

Supplementary Figure 5, Almozyan S et al.

Supplementary Figure 5. The effect of cisplatin on FLYWCH1 protein expression in HCT116 and SW480 cell lines treated with 16µM of cisplatin, and CHX or CHX/MG132 at different time point. WB analysis confirms the steady expression of FLYWCH1 in HCT116 cells (left panel), while cisplatin treatment reduces protein level possibly via proteasome degradation pathway, examined by absent (middle panel) or present (right panel) of proteasome inhibitor MG132 in SW480 cells. Cells were The cells were incubated with actinomycin D for 1 hr to inhibit RNA synthesis, and then treated with 0.1mM CHX and 10υM MG132 at different time points. 100µg of protein lysate was loaded per well, and protein loading levels are monitored by probing for β-actin.

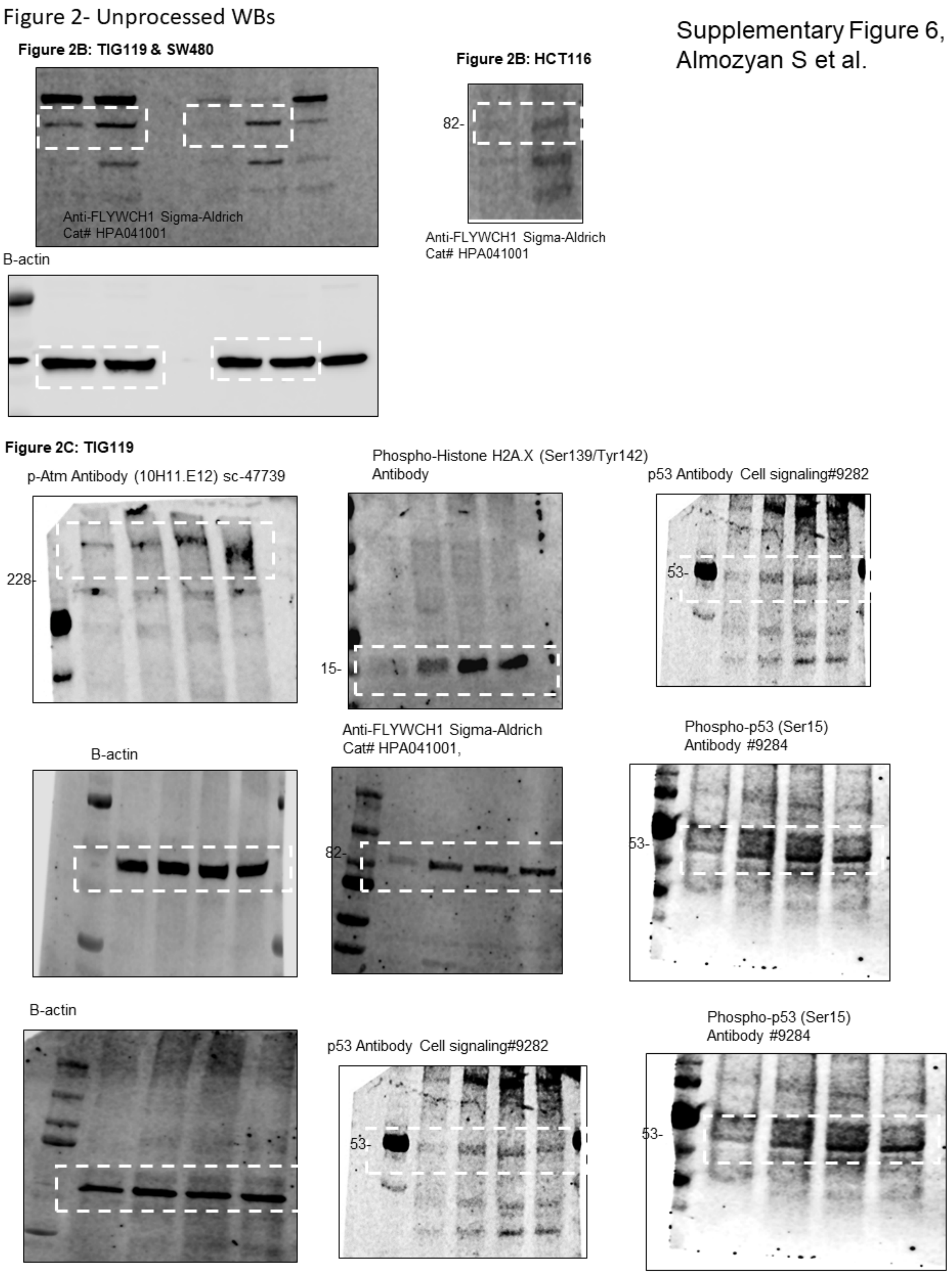

Supplementary Figure 6. Unprocessed WBs used in Figure 2.

Figure 3- Unprocessed WBs

Supplementary Figure 7,
Almozyan S et al.

Figure 3B

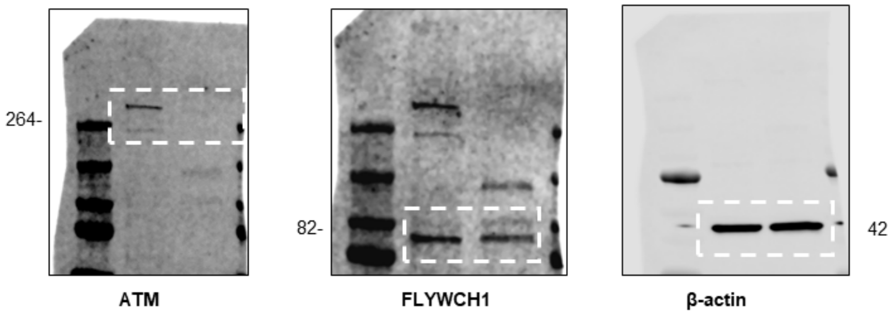

Figure 3G

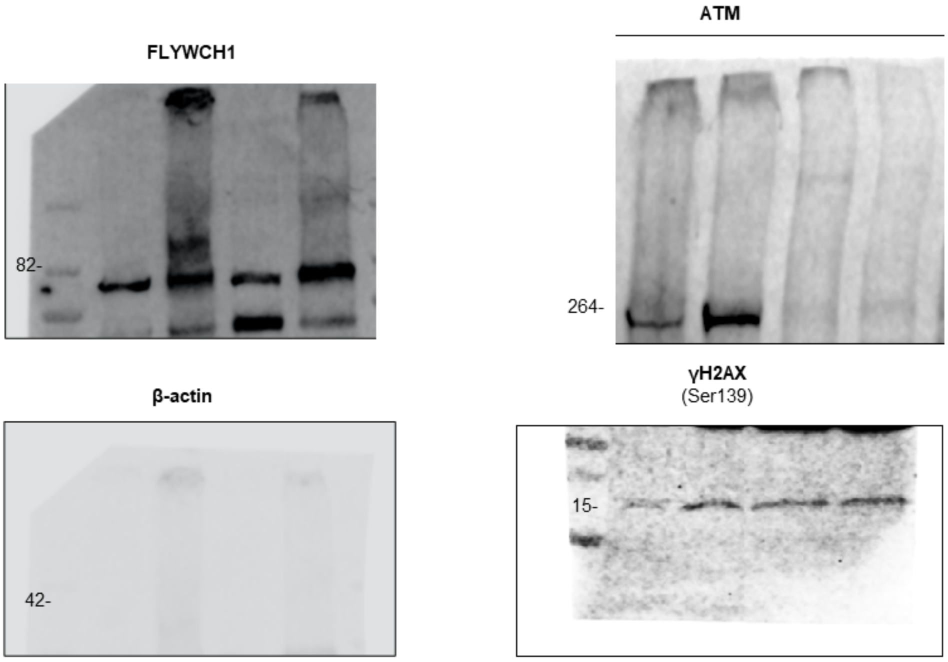

Supplementary Figure 7. Unprocessed WBs used in Figure 3.

Figure 4- Unprocessed WBs

Supplementary Figure 8, Almozyan S et al.

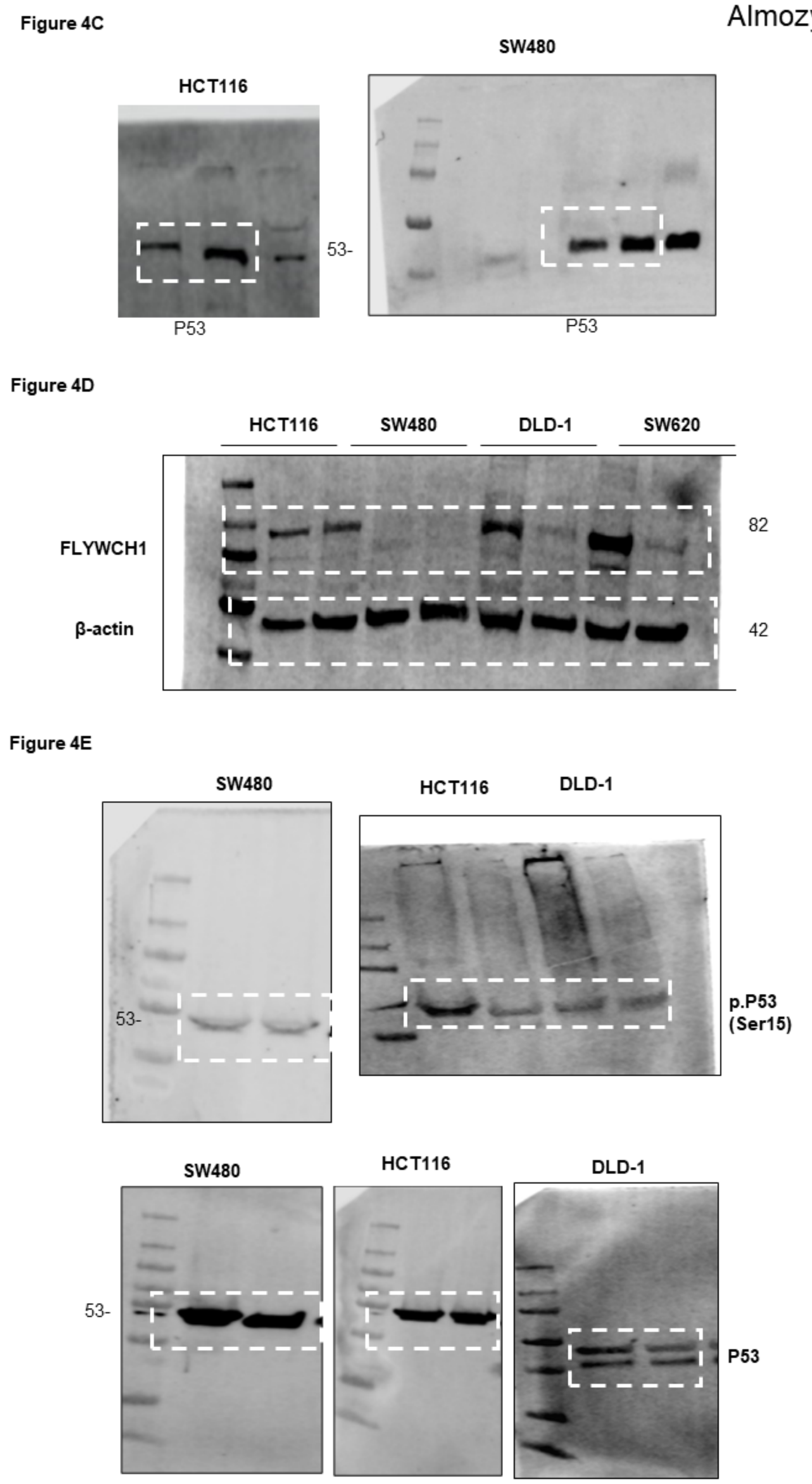

Supplementary Figure 6. Unprocessed WBs used in Figure 4.
